# Active involvement of patients, radiation oncologists, and surgeons in a multidisciplinary team approach: Guiding local therapy in recurrent, metastatic rectal cancer

**DOI:** 10.1002/cam4.6667

**Published:** 2023-11-01

**Authors:** Seo Hee Choi, Gowoon Yang, Woong Sub Koom, Seung Yoon Yang, Seung‐Seob Kim, Joon Seok Lim, Han Sang Kim, Sang Joon Shin, Jee Suk Chang

**Affiliations:** ^1^ Department of Radiation Oncology Yonsei University College of Medicine Seoul Korea; ^2^ Department of Surgery Yonsei University College of Medicine Seoul Korea; ^3^ Department of Radiology Yonsei University College of Medicine Seoul Korea; ^4^ Division of Medical Oncology, Department of Internal Medicine Yonsei University College of Medicine Seoul Korea

**Keywords:** decision making, multidisciplinary team, oligometastasis, patient outcomes, rectal cancer, salvage therapy

## Abstract

**Background:**

Despite the extensive implementation of an organized multidisciplinary team (MDT) approach in cancer treatment, there is little evidence regarding the optimal format of MDT. We aimed to investigate the impact of patient participation in MDT care on the actual application rate of metastasis‐directed local therapy.

**Methods:**

We identified all 1211 patients with locally advanced rectal cancer treated with neoadjuvant radiochemotherapy at a single institution from 2006 to 2018. Practice patterns, tumor burden and OMD state were analyzed in recurrent, metastatic cases.

**Results:**

With a median follow‐up of 60.7 months, 281 patients developed metastases, and 96 (34.2%), 92 (32.7%), and 93 (33.1%) patients had 1, 2–5, and >5 lesions, respectively. In our study, 27.1% were managed in the MDT clinic that mandated the participation of at least four to five board‐certified multidisciplinary experts and patients in decision‐making processes, while the rest were managed through diverse MDT approaches such as conferences, tumor board meetings, and discussions conducted via phone calls or email. Management in MDT clinic was significantly associated with more use of radiotherapy (*p* = 0.003) and more sessions of local therapy (*p* < 0.001). At the time of MDT clinic, the number of lesions was 1, 2–5, and >5 in 9 (13.6%), 35 (53.1%), and 19 (28.8%) patients, respectively. The most common states were repeat OMD (28.8%) and de novo OMD (27.3%), followed by oligoprogression (15%) and induced OMD (10.6%).

**Conclusion:**

Our findings suggest that active involvement of patients and radiation oncologists, and surgeons in MDT care has boosted the probability of using local therapies for various types of OMD throughout the course of the disease.

## 1 INTRODUCTION

Colorectal cancer (CRC) ranks as the second and third most prevalent cancer in women and men, respectively, and it accounts for nearly 0.7 million deaths globally every year.^1^ More than 20% of CRC patients present with metastatic disease at diagnosis, and nearly half of nonmetastatic patients who receive curative treatment will eventually present with metastatic disease.^2^ Furthermore, approximately 20%–30% of rectal cancer patients present with metastatic disease at diagnosis, with the most common sites of metastases being the liver, lungs, and distant lymph nodes.^3^, ^4^ The oligometastatic disease (OMD) theory proposes a distinct state between localized and systemically metastasized disease.^5^ Early studies showed increased survival when metastasis‐directed local therapy is added to standard systemic therapy for OMD.^6^, ^7^, ^8^, ^9^ As the separation between curative and palliative intent in local therapy becomes less distinct, making local treatment decisions at the individual patient level becomes increasingly complicated, especially in the context of fundamentally incurable disease.

Multidisciplinary team (MDT) management offer a forum for a multidisciplinary staff, which includes surgeons, radiologists, medical oncologists, and radiation oncologists, to discuss the diagnostic and therapeutic approaches to patients periodically as part of routine cancer care pathways.^10^ Consequently, MDT implementation can centralize expertise, and optimal outcomes are observed in centers treating the highest patient volume under MDT pathways.^11^ Despite the crucial importance of clear communication about the benefits and risks associated with local therapy for informed patient decision‐making, there is scant evidence and consensus about the optimal format, whether it be tumor board meetings, discussions via telephone calls or email, or MDT clinics involving patients.^12^

Prior to 2012, our MDT approach included conferences, consultations, emails, and phone discussions. In 2012, we launched a MDT clinic that requires mandatory patient participation and involves a minimum of four to five board‐certified multidisciplinary physicians in the decision‐making process. However, due to the absence of clear consensus, MDT clinic was used at the clinician's discretion and various methods of MDT approaches, including conferences, consultations, and emails or phone discussions, were utilized in the clinic for the management of recurrent and metastatic CRC. Within this context, we investigated the impact of patient participation in MDT care on the actual application rate of metastasis‐directed local therapy and overall survival in an unselected, population‐based institutional cohort.

## 2 METHODS

### 2.1 Study

Using a prospectively maintained database, we identified consecutive patients with newly diagnosed locally advanced rectal cancer (LARC) who were treated with neoadjuvant concurrent chemoradiotherapy (CCRT) at a single institution between 2006 and 2018. Medical charts of 1390 patients who underwent preoperative radiotherapy for LARC were reviewed. Patients who presented with de novo stage IV rectal cancer, those with histology other than adenocarcinoma, or patients lost to follow‐up within 3 months were excluded.

A total of 1211 consecutive patients with available medical records and images for review were included in the study. Among these patients, a total of 281 patients who were diagnosed with metachronous, recurrent, or metastatic disease from primary rectal cancer following initial curative treatment formed recurrence subset. For each patient, baseline characteristics at the time of initial diagnosis of LARC and the detection of recurrent/metastatic disease from rectal cancer were reviewed. In recurrence subset, computed tomographic (CT) scan, magnetic resonance imaging (MRI), and/or positron emission tomography (PET) images were reviewed if available. The type of recurrence was classified as follows according to the number of metastases on images regardless of whether they were present in the same organ or not: 1 metastasis, 2–5 metastases, and >5 metastases. Cases with carcinomatosis or peritoneal lesions were classified as having >5 metastases. In patients with MDT, the reasons for referral, disease status according to a consensus recommendation for classification of OMD by the European Society for Radiotherapy and Oncology (ESTRO) and the European Organisation for Research and Treatment of Cancer (EORTC),^13^ information on salvage treatments, and follow‐up data were also reviewed. The study was approved by the institutional review board of our institute (4–2021‐1222), and all procedures were carried out in accordance with relevant guidelines and regulations. Since the study was retrospective, the need for written informed consent was waived.

### 2.2 Multidisciplinary team

Prior to 2012, our MDT approach consisted of conferences, consultations, emails, and phone discussions. In 2012, we launched an MDT clinic (MDT‐pt) that encourages mandatory participation of patient and at least four to five board‐certified multidisciplinary physicians in the decision‐making process. However, the decision to discuss a patient through conferences, tumor board meetings, emails/phones, or the MDT‐pt clinic was left to the physician's discretion. The MDT conference and tumor board meetings are conducted weekly, while the MDT‐pt clinic is scheduled based on demand. In both formats, discussions involve a thorough review of the patient's history and imaging, along with discussions on potential local or systemic treatment options. In the MDT‐pt clinic, an effort is made to reach team consensus through active interdisciplinary discussion prior to patient involvement. The goal of the MDT‐pt clinic is to foster clear and compassionate communication between physicians and patients. This involves discussing the benefits and risks associated with therapies to ensure that patients make informed treatment choices, guided by evidence‐based, personalized advice from the MDT that aligns with the individual's values and goals.

In this study, patients with MDT were defined as those with a medical claim for the MDT clinic and for whom patient‐specific treatment decisions were individualized and shared through effective communication with the MDT clinic. Characteristics of patients according to the year of diagnosis (pre‐MDT era 2006–2011: “pre‐MDT clinic cohort” versus MDT era 2012–2018: “MDT clinic cohort”) are summarized in Table S1. During the study period, a total of 1948 CRC‐MDT clinic encounters were identified based on medical claims. In recurrence subset, 66/281 patients with recurrent/metastatic disease were managed through MDT clinics.

### 2.3 Treatment for recurrent/metastatic disease

For patients with a low tumor burden of recurrent/metastatic disease, upfront surgical resection was generally preferred. By contrast, for patients with borderline/unresectable disease, local therapy was generally deferred until the disease burden was mitigated after upfront systemic therapy.^14^, ^15^, ^16^ The use of metastasis‐directed radiotherapy either through conventional fractionations or stereotactic ablative methods was increasingly discussed over time in various clinical scenarios as an adjunct or alternative to surgical treatments in the following cases: patients who refused surgery, patients who were not candidates for surgery,^14^, ^17^ symptomatic patients, cases of re‐irradiation,^18^, ^19^ and cases of oligo‐progression^20^ which presents as a few progressing lesions on a background of widespread stable metastatic disease (Figure S1).

There were important considerations for deciding whether, when, and how to integrate local therapies, including (1) disease location, burden, and rate of progression, (2) patient's health condition and willingness to accept additional risk of local therapy, and (3) potential for minimal interruption of subsequent systemic therapy after completion of local therapy. Patient‐specific discussions continued over the entire follow‐up period.

In the current study, local therapy was defined as metastasectomy or metastasis‐directed radiotherapy. Systemic therapies were applied based on national clinical practice guidelines for recurrent/metastatic rectal cancer patients.^21^ FOLFOX (oxaliplatin, infusional fluorouracil, and leucovorin) or FOLFIRI (irinotecan, infusional fluorouracil, and leucovorin) were selected as first‐line treatment. Bevacizumab (Avastin) or cetuximab (Erbitux) are also added based on the presence of KRAS or BRAF mutations. Capecitabine (Xeloda), trifluridine/tipiracil (TAS‐102; Lonsurf), or regorafenib (Stivarga) were used as next‐line treatments.

### 2.4 Data analysis

**FIGURE 1:**
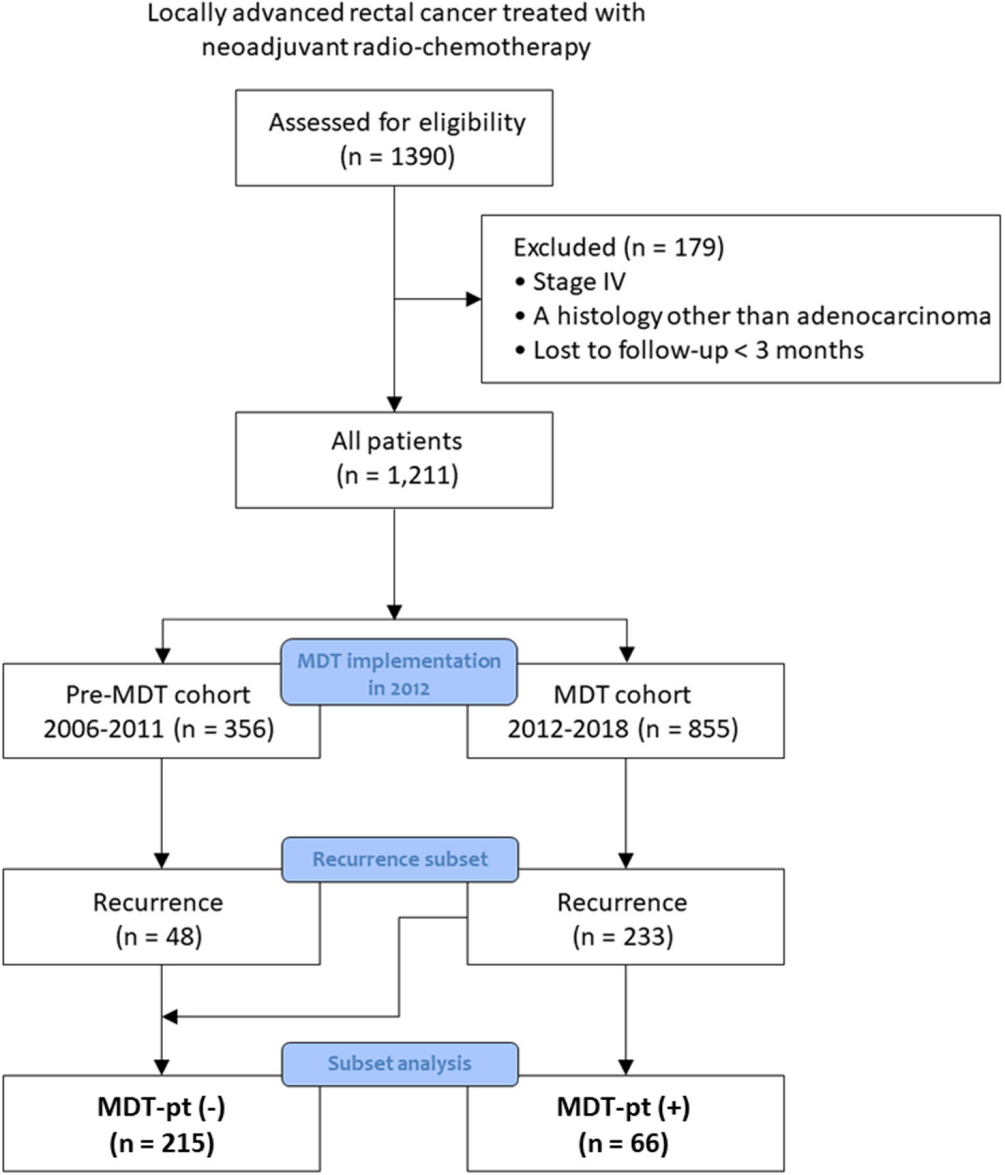
Patient flow diagram through the different phases of the study.

We divided the patients with recurrence into two groups based on the patient's participation in MDT clinic: (1) MDT‐pt (+) group, which included patients with at least one referral to MDT clinic for patient‐specific treatment discussion and (2) MDT‐pt (−) group, which included all the other patients not belonging to the first group (Figure 1). The initial disease and treatment characteristics and disease burden (number of diseases and serum level of carcinoembryonic antigen [CEA]) at first recurrence were compared between the two groups using *t*‐test and chi‐squared test. Secondly, we evaluated the disease state at the time of MDT referral according to the ESTRO/EORTC OMD classification and investigated whether MDT accelerated the use of local therapy with broader indications within multiple and different states of OMD throughout a patient's disease course. Then, we analyzed the benefit of local therapy (surgery and/or radiotherapy) on overall survival (OS) from the date of first recurrence using univariate and multivariate Cox regression models. Finally, within all patients, recurrence‐free survival (RFS), which is defined as the time from initial diagnosis to cancer recurrence, all‐cause death, or last follow‐up, and OS, which is defined as a length of survival following initial diagnosis to all‐cause death, or last follow‐up were compared according to the year of initial diagnosis (pre‐MDT era 2006–2011: “pre‐MDT clinic cohort” versus MDT era 2012–2018: “MDT clinic cohort”).

The Kaplan–Meier method with log‐rank test was used to estimate survival rates. The median follow‐up was calculated in both groups by means of the reverse Kaplan–Meier method.^22^ One‐way ANOVA test with post hoc analysis using the Bonferroni test was used to compare acquired values among subgroups. Cox regression analysis using proportional hazards modeling was used in multivariate analyses. The proportional hazards assumption was verified using the Schoenfeld Residuals Test. When the hazard for a variable was suspected to be nonproportional over time, we built a time‐dependent Cox regression model, a nonproportional hazard model. Statistical significance was set at a *p* value <0.05. Statistical analysis was performed using R version 4.3.1 (R Foundation for Statistical Computing, Vienna, Austria) and IBM SPSS statistics, version 25.0 (SPSS Inc., Chicago, IL).

## 3 RESULTS

The study population consisted of 1211 patients with LARC treated with neoadjuvant CCRT and total mesorectal excision. With the median follow‐up of 60.7 months (range 4.1–320.2), 281 patients (23.2%) developed distant recurrences with (*n* = 55) or without (*n* = 226) local recurrence. When classified by number of lesions at first recurrence, 96 (34.2%), 92 (32.7%), and 93 (33.1%) patients had 1, 2–5, and >5 lesions, respectively.

Patients were categorized into MDT‐pt (+) group (*n* = 66) or MDT‐pt (−) group (*n* = 215), as 48 patients in the pre‐MDT clinic cohort were included in MDT‐pt (−) group (Figure 1). The comparison results of characteristics between the pre‐MDT clinic cohort and the MDT clinic cohort in patients with recurrences are shown in Table S2. The median follow‐up times from the initial diagnosis were 68.0 months for the MDT‐pt (+) group and 65.6 months for the MDT‐pt (−) group, respectively (*p* = 0.410). Additionally, the median follow‐up times from the first recurrence were 48.2 months for the MDT‐pt (+) group and 47.6 months for the MDT‐pt (−) group, respectively (*p* = 0.419). In MDT‐pt (+) group, an average of 1.6 ± 0.0 MDT clinics was held per patient (range, 1–6) after an average of 14.1 ± 2.2 months (median 6.6 months) from the first recurrence. Thirty‐two percent of patients (*n* = 21) was referred to MDT clinic within 1 month. Referral to MDT clinics rate steadily increased from 11.1% to 27.1% between 2012 and 2018. There were no significant differences in baseline characteristics at the time of either initial treatment or recurrence (all *p* > 0.05), including initial disease burden (1, 2–5 vs. >5) and serum CEA level (Table 1). At the time of MDT‐pt, number of lesions were 1, 2–5, and >5 in 9 (13.6%), 35 (53.1%), and 19 (28.8%) patients, respectively (Table 2). The most common state was repeat OMD state (28.8%) and de novo OMD state (27.3%). Fifteen percent of patients was oligoprogression and 10.6% was induced OMD state which had a few active lesions after most lesions resolved or controlled by systemic therapy with a history of widespread metastases. In 19 patients with >5 metastatic lesions, 17 were discussed for the potential use of local therapy. Compared to MDT‐pt (−) group, MDT‐pt was associated with more local therapy use (63.6% vs. 42.8%, *p* = 0.003) and higher cumulative number of local therapy use (surgery and/or radiotherapy; 2.6 ± 2.0 vs. 1.6 ± 1.6, *p* < 0.001) (Table 3). In MDT‐pt (−) group, the number of local therapy use did not increase significantly over time (Table S3). The differences in OS based on the first recurrence pattern, MDT‐pt status, and recurrence pattern at MDT‐pt are presented in Figure S2. The patients who had polymetastases at first recurrence but were in the OMD state when participating in the MDT discussion showed the highest OS rate (5‐year OS 100% vs.28.6% (polymetastases at both first recurrence and MDT‐pt; *p* = 0.042) vs. 37.1% (polymetastases at first recurrence, MDT‐pt (−); *p* = 0.015)).

**TABLE 1:** Comparison of characteristics according to the active involvement of patients in MDT discussions and decision‐making processes (MDT‐pt) within recurrence subset (*n* = 281).

		MDT‐pt (+) group (*n* = 66)		MDT‐pt (−) group (*n* = 215)		*p* Value^c^
		No.	%	No.	%	
Age (median [range], years)^a^		60 (36–83)		60 (24–88)		0.802
Pathologic stage^b^	Stage 0	3	4.5	8	3.7	0.290
	Stage 1	14	21.2	30	14.0	
	Stage 2	15	22.7	72	33.5	
	Stage 3	34	51.5	105	48.8	
Tumor location^b^	Upper‐Middle	40	60.6	128	59.5	0.877
	Lower	26	39.4	87	40.5	
Adjuvant chemotherapy^b^	Yes	43	65.2	144	67.0	0.783
	No	23	34.8	71	33.0	
Number of tumor/metastatic lesion^a^	1	19	28.8	77	35.8	0.260
	2–5	27	40.9	65	30.2	
	>5	20	30.3	73	34.0	
CEA (median (range), ng/mL)^a^		4.0 (0.7–235.8)		3.3 (0.4–1293)		0.455
	Normal	23	34.8	87	40.5	0.413
	Elevated	43	65.2	128	59.5	

^a^ At the time of recurrence.

^b^ At the time of initial primary treatment for locally advanced rectal cancer.

^c^ A *t*‐test was used for comparison of continuous variables, and Pearson's Chi‐square test was used for comparison of noncontinuous variables between the two groups.

Abbreviations: CEA, carcinoembryonic antigen; MDT, multidisciplinary team.

**TABLE 2:** Details of the disease burden and disease states at the time of patients' involvement in MDT discussions (MDT‐pt) (*n* = 66).

Pattern of failures (at the time of MDT‐pt)	At the first recurrence					
	1 lesion		2 ~ 5 lesions		>5 lesions	
	No.	%	No.	%	No.	%
[According to the disease burden]						
Unclassified^a^	0	0.0	3	11.1	0	0.0
1 lesion	7	36.8	1	3.7	1	5.0
2 ~ 5 lesions	9	47.4	20	74.1	6	30.0
>5 lesions	3	15.8	3	11.1	13	65.0
[According to ESTRO/EORTC OMD classification]						
Synchronous oligometastasis	0	0.0	0	0.0	0	0.0
De novo oligorecurrence	6	37.5	10	47.6	0	0.0
De novo oligoprogression	1	6.3	1	4.8	0	0.0
Repeat oligorecurrence	3	18.8	2	9.5	0	0.0
Repeat oligopersistence	3	18.8	4	19.0	0	0.0
Repeat oligoprogression	3	18.8	4	19.0	0	0.0
Induced oligorecurrence	0	0.0	0	0.0	2	28.6
Induced oligopersistent	0	0.0	0	0.0	4	57.1
Induced oligoprogression	0	0.0	0	0.0	1	14.3

Abbreviations: EORTC, European Organization for Research and Treatment of Cancer; ESTRO, European Society for Radiotherapy and Oncology; MDT, multidisciplinary team; OMD, oligometastatic disease.

^a^ There were no obvious recurrent lesions and diagnostic aspects was discussed.

**TABLE 3:** Practice patterns of local therapy (radiotherapy and/or surgery) usage according to patients' involvement in MDT discussions (MDT‐pt) within recurrence subset (*n* = 281).

	MDT‐pt (+) group (*n* = 66)	MDT‐pt (−) group (*n* = 215)	*p* value
In all patients			
Percentage who received ≥1 RT	63.6%	42.8%	0.003
Percentage who received ≥1 surgery	72.7%	63.7%	0.177
Percentage who received ≥1 surgery or RT	84.8%	78.1%	0.236
Cumulative number of RT session per patient	1.3 ± 1.5	0.7 ± 1.2	0.004
Cumulative number of surgery session per patient	1.3 ± 1.1	0.9 ± 1.0	0.007
Cumulative number of surgery ± RT session per patient	2.6 ± 2.0	1.6 ± 1.6	<0.001
Cumulative number of changes in systemic therapy regimens	2.5 ± 1.3	1.7 ± 1.2	<0.001
Percentage who received FOLFOX or FOLFIRI	87.9%	77.8%	0.072
Percentage who received Capecitabine	57.6%	36.6%	0.002
Percentage who received Trifluridine/tipiracil	4.5%	2.8%	0.475
Percentage who received targeted agent	68.2%	54.2%	0.044
In patients with 1–5 metastases at first recurrence			
Percentage who received ≥1 RT	63%	43.7%	0.022
Percentage who received ≥1 surgery	82.6%	76.1%	0.354
Percentage who received ≥1 surgery or RT	91.3%	89.4%	0.715
Cumulative number of RT session per patient	1.3 ± 1.6	0.7 ± 1.1	0.029
Cumulative number of surgery session per patient	1.5 ± 1.1	1.1 ± 1.0	0.017
Cumulative number of surgery ± RT session per patient	2.8 ± 1.8	1.8 ± 1.6	0.001
In patients with >5 metastases at first recurrence			
Percentage who received ≥1 RT	65%	41.1%	0.057
Percentage who received ≥1 surgery	50%	39.7%	0.409
Percentage who received ≥1 surgery or RT	70%	56.2%	0.265
Cumulative number of RT session per patient	1.4 ± 1.5	0.7 ± 1.2	0.034
Cumulative number of surgery session per patient	0.9 ± 1.1	0.5 ± 0.8	0.151
Cumulative number of surgery ± RT session per patient	2.3 ± 2.3	1.3 ± 1.7	0.065

Abbreviations: MDT, Multidisciplinary team; RT, radiotherapy.

Uni‐ and multi‐variable Cox regression models demonstrated that use of local therapy (surgery and/or radiotherapy) was independently associated with longer OS along with disease burden (1–5 vs. >5 lesions), serum CEA level, and total lines of chemotherapy regimens (Table 4). The 5‐year OS rate for the patients with 1–5 metastases was 74.0% with local therapy and 49.1% without local therapy. In the patients with >5 metastases, the 3‐year OS rate with or without local therapy was 55.4% and 35.8%, respectively.

**TABLE 4:** Univariate and multivariate Cox regression models for overall survival (from disease recurrence) within recurrence subset (*n* = 281).

Variables	Univariate analysis			Multivariate analysis		
	HR	95% CI	*p* Value	HR	95% CI	*p* Value
Age (continuous)^a^	1.02	1.00–1.03	0.118	1.02	0.99–1.05	0.062
Sex (Female vs. Male)^b^	0.80	0.48–1.31	0.364	0.63	0.36–1.09	0.097
Stage (continuous)^b^	1.03	0.79–1.33	0.846	1.63	0.94–2.83	0.084
Tumor location (lower vs. upper‐mid)^b^	0.91	0.48–1.73	0.775	0.86	0.44–1.66	0.651
Pathologic complete response after preoperative CCRT^b^	0.85	0.27–2.69	0.780	1.19	0.37–3.83	0.774
First recurrence type (Oligo‐, 1–5 vs. polymetastasis, >5)^a^	0.28	0.18–0.44	<0.001	0.38	0.23–0.63	<0.001
Use of local therapy (surgery and/or RT; Yes vs. No)^a^	0.25	0.15–0.41	<0.001	0.49	0.26–0.90	0.022
CEA (continuous)^a^	1.01	1.00–1.01	<0.001	1.00	1.00–1.01	<0.001
Total lines of chemotherapy (continuous)^a^	1.01	1.00–1.01	0.001	1.01	1.00–1.02	0.002
Use of targeted agents (bevacizumab or cetuximab)^a^	1.01	0.99–1.03	0.186	0.99	0.98–1.02	0.642

Abbreviations: CCRT, concurrent chemoradiotherapy; CEA, carcinoembryonic antigen; CI, confidence interval; HR, hazard ratio; RT, radiotherapy.

^a^ At the time of recurrence.

^b^ At the time of initial primary treatment for locally advanced rectal cancer.

**FIGURE 2:**
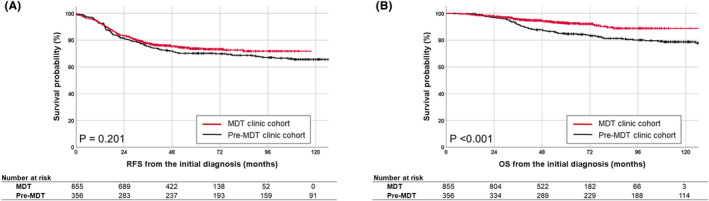
Kaplan Meier survival curves for (A) recurrence‐free survival (RFS) from the initial diagnosis and (B) overall survival (OS) from the initial diagnosis according to the year of initial diagnosis and treatment: Pre‐MDT clinic cohort (2006–2011) versus MDT clinic cohort (2012–2018).

Among all 1211 patients, the median follow‐up times from the date of initial diagnosis in the pre‐MDT clinic cohort and the MDT clinic cohort were 98.9 months and 56.3 months, respectively. Characteristics of these two groups are shown in Table S1. The 5‐year RFS was not significantly differed by the date of diagnosis (2006–2011, 70.2% vs. 2012–2018, 73.6%, *p* = 0.201, Figure 2A). The 5‐year OS was significantly longer in the 2012–2018 vs. 2006–2011 (92.8% vs. 84.7%, *p* < 0.001, Figure 2B).

## 4 DISCUSSION

While trials are ongoing to determine the survival benefits of local therapy in OMD, its implementation has inherent challenges. These include evaluating the feasibility of local control—whether visible metastases can be resected or managed with various modalities—and aligning patients' and physicians' expectations about the realities of incorporating local therapy in fundamentally incurable conditions. In this large, population‐based cohort study, we noted that active participation from both patients and local therapists was associated with an increased use of local therapies, which potentially improved survival. The active participation of local therapists in MDT discussions amplified the focus on identifying not only genuine OMD states, but also repeat or induced OMD states, and selected polymetastatic disease states. Moreover, the complexity increases for patients with multiple disease progressions considering repeated local therapies. With limited evidence for these therapies' long‐term effects on survival, it is essential to maintain clear team communication with patients for individualized treatment adjustments based on ongoing feedback.

Christ et al. and Galata et al. reported that MDTs had resulted in an increased referral for local therapy in 47% and 69% of OMD cases, respectively.^12^, ^23^ Other studies also reported that overall use of radiotherapy and chemotherapy were significantly increased in the MDT cohort.^24^, ^25^ In our study, the majority of patients (89.9%), excluding those with near complete response to systemic therapy, underwent at least one course of local therapy, and both groups were associated with considerably higher 5‐year OS than that reported in the literature (local therapy yes vs. no; 74% vs. 49.1%, *p* = 0.103). In light of around 80% of metastatic CRC patients being deemed unresectable at diagnosis,^26^ we postulate that the key advantage of MDT could be the dynamic reassessment of tumor burden for optimal timing of local therapy, a process possibly fostered by enhanced patient engagement.

In the present study, despite widespread active use of MDT clinics in colorectal cancer (1948 encounters between 2012 and 2018), active participation of patients and various experts via MDT clinic was underutilized in recurrent/metastatic rectal cancer (66/233) during the same study period and other patients were discussed outside the MDT clinics (e.g., email, phone call, and conference). This could occur if physicians, based on prior experiences, exclude local therapy without a local therapist's input, or if they assume patients are uninterested in local therapy, potentially neglecting its consideration as a viable option.

The liver is the most common site of metastases in CRC. With the advances in surgical techniques, such as a two‐step approach, portal vein embolization, and combined ablation and resection, local therapy has become a standard of care and expanded the definition of resectability.^27^ Moreover, local therapy for other sites of metastases, including lung, lymph nodes, pelvic cavity, bone, and brain, is increasingly being used and has been reported to be effective not only in symptom palliation but also in life prolongation in multiple, nonrandomized studies.^28^ As treatment sites include more than one site, the formulation of local treatment strategy becomes more complicated in terms of consensus on who, where, what, when, and how. Because of the difficulty in conducting histology‐specific and treatment site‐specific clinical trials,^29^ treatment indications and key considerations are normally extrapolated from experiences of liver metastatic CRC. Considering prior reports that suggest changes in surgery type or resection order often occur post‐MDTs,^24^, ^30^ it is evident that further research is needed to optimally structure MDT clinics, mandating patient and local therapist involvement for appropriate indication and timing, all while avoiding excessive workload.

Better OS was associated with the MDT era cohort despite similar RFS, aligning with previous smaller series findings,^24^, ^25^, ^31^, ^32^, ^33^, ^34^ yet these results warrant cautious interpretation. Other potential explanations could include advances in systemic therapy management, unidentified confounding factors, or unique patterns of disease progression and tumor biology suggested by Willmann et al.^35^ Interestingly, our findings about the impact of local therapy and MDT on patients with >5 metastases align with our previous study involving 4157 metastatic CRC patients.^20^ That study showed metastasis‐directed radiotherapy delayed changes in next‐line systemic therapy in patients with oligoprogressive disease following polymetastasis, which implies the role of local therapy in managing patients with a larger metastatic tumor burden should be an active area of research.^36^

This study has several limitations, including the potential risk of residual confounding. To minimize confounding variables and patient heterogeneity, we limited the inclusion criteria to “LARC requiring neoadjuvant CCRT” and “patients who had undergone the same follow‐up schedule.” Although MDT was utilized at the clinician's discretion, there was no significant difference in the MDT referral rate among physicians (data not shown). Furthermore, we were unable to provide information about the patient's performance status at MDT‐pt or the effect of local treatment on patient‐related outcomes. More studies involving a larger number of patients would be helpful in addressing these aspects more comprehensively.

In our population‐based cohort study, we found that active involvement of patients and radiation oncologists, and surgeons in MDT care enhanced the likelihood of using local therapies for various types of OMD throughout the course of the disease. This finding could play a significant role in transferring the progression‐free survival advantage observed in multiple phase II trials to patients treated in everyday clinical practice, but efforts are required to utilize MDT easily and more efficiently considering the potential increase in physicians' workload with a regular MDT in metastatic patients.^12^ As shown in our previous work,^37^ artificial intelligence‐based tools would enable a quick estimation of disease burden and identification of OMD state, thus increasing their chance of being presented in MDTs and eventually being referred for local therapy Additionally, CRC histology‐ and site‐specific phase III trials are needed to guide our practice of local therapy.

## AUTHOR CONTRIBUTIONS

**Seo Hee Choi:** Conceptualization (equal); data curation (equal); formal analysis (equal); investigation (equal); methodology (equal); writing – original draft (equal); writing – review and editing (equal). **Gowoon Yang:** Data curation (lead); methodology (equal); resources (equal). **Woong Sub Koom:** Conceptualization (equal); resources (equal); supervision (equal); validation (equal). **Seung Yoon Yang:** Investigation (equal); resources (equal); supervision (equal). **Seung‐Seob Kim:** Investigation (equal); methodology (equal); supervision (equal). **Joon Seok Lim:** Conceptualization (equal); supervision (equal); validation (equal); visualization (equal). **Han Sang Kim:** Conceptualization (equal); formal analysis (equal); supervision (equal); validation (equal). **Sang Joon Shin:** Conceptualization (equal); project administration (equal); resources (equal); supervision (equal); validation (equal). **Jee Suk Chang:** Conceptualization (lead); formal analysis (lead); investigation (equal); project administration (equal); supervision (lead); validation (equal); visualization (equal); writing – original draft (equal); writing – review and editing (lead).

## FUNDING INFORMATION

This research was supported by the National Research Foundation of Korea (NRF) grant funded by the Korean government (MSIT) (NRF‐2021R1A2C1010900, NRF‐2022R1F1A1074344, and NRF‐2021R1F1A1055641) and a grant of the Korea Health Technology R&D Project through the Korea Health Industry Development Institute (KHIDI), funded by the Ministry of Health & Welfare, Republic of Korea (grant number: HI21C0974). The funder had no role in the design and conduct of the study; collection, management, analysis, and interpretation of the data; preparation, review, or approval of the manuscript; and decision to submit the manuscript for publication.

## Supporting information


Appendix S1

## Data Availability

Data are available upon reasonable request. Data from the study are available upon request from the corresponding author (changjeesuk@yuhs.ac). Data sharing will only be available for academic research, but not for other objectives (i.e., commercial use). A data use agreement will be required before the release of data and institutional review board approval, as appropriate.
