# Supplementary material for: Active involvement of patients, radiation oncologists, and surgeons in a multidisciplinary team approach: Guiding local therapy in recurrent, metastatic rectal cancer
Source: Cancer Med. 2023 Nov 1;12(22):21057–67. doi: 10.1002/cam4.6667 (PMC10709736; doi:10.1002/cam4.6667)
Supplement: Supplementary file 1 — Appendix S1 [file CAM4-12-21057-s001.docx]

**Supplementary Figure 1.** The number of cases in our department which local radiotherapy (RT) was performed for metastatic lesions of rectal cancer, according to each year

3D RT, 3-dimensional radiotherapy; IMRT, intensity-modulated radiotherapy; SBRT, stereotactic body radiation therapy

**Supplementary Figure 2.** Kaplan Meier survival curves for overall survival (OS) from the disease recurrence according to the pattern of failures at first recurrence (a) in patients with oligometastatic disease (OMD) at first recurrence, (b) in patients with polymetastases (PM) at first recurrence, and (c) in patients with PM at first recurrence, when stratified based on MDT participation or patterns of failure at MDT clinic

**
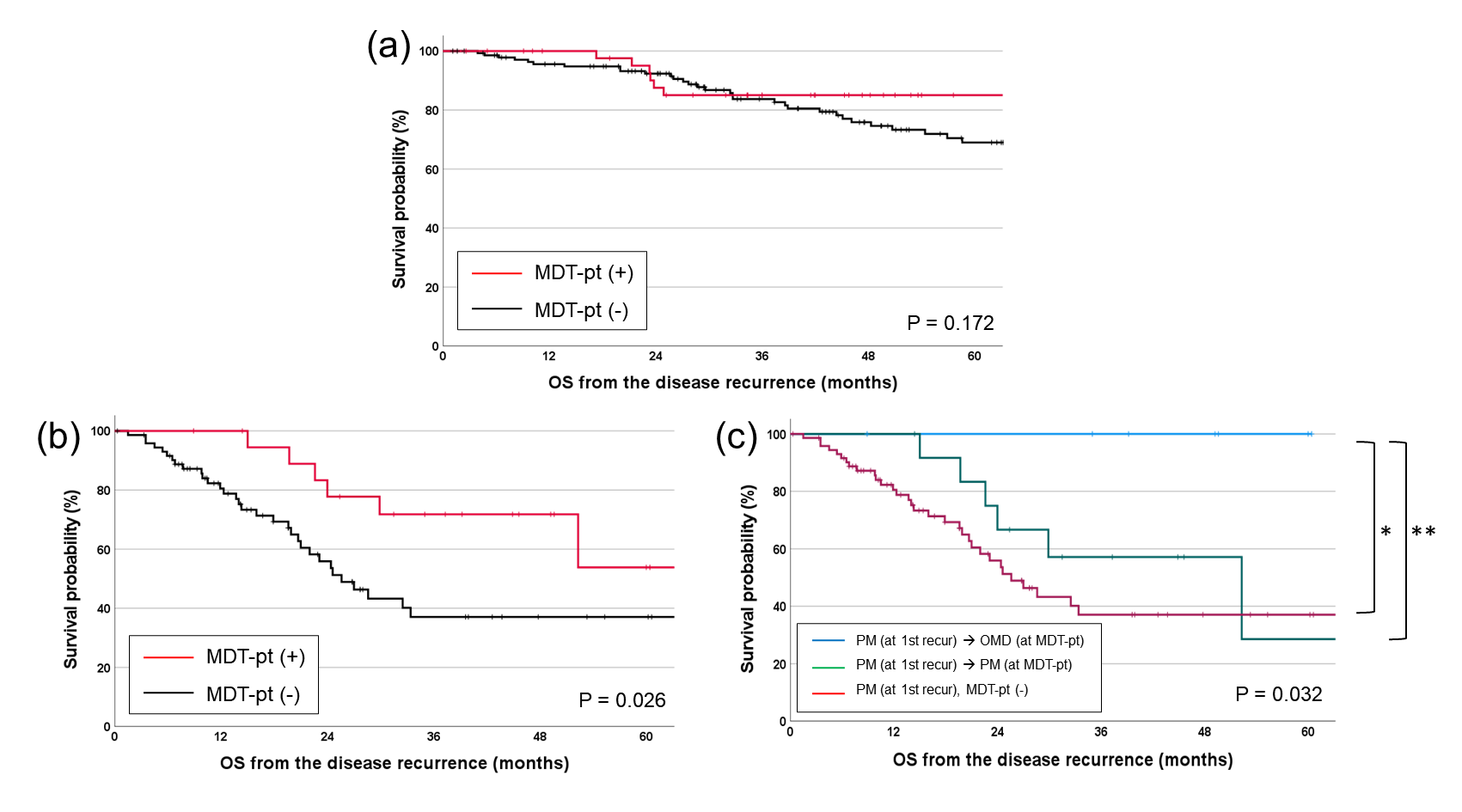
**

PM (at 1st recur) -> OMD (at MDT-pt) vs. PM (at 1st recur) -> PM (at MDT-pt): P = 0.042

PM (at 1st recur) -> OMD (at MDT-pt) vs. PM (at 1st recur), MDT-pt (-): P = 0.015

PM (at 1st recur) -> PM (at MDT-pt) vs. PM (at 1st recur), MDT-pt (-): P = 0.290

(P-values less than 0.05 were indicated as * or **)

**Supplementary Table 1.** Baseline characteristics of 1,211 patients with locally advanced rectal cancer according to the year of diagnosis (pre-MDT era 2006-2011: “pre-MDT clinic cohort” vs MDT era 2012-2018: “MDT clinic cohort”)

|  |  |  |  | **Pre-MDT clinic cohort**  **(n = 356)** | | **MDT clinic cohort**  **(n = 855)** | |  |  |  |
| --- | --- | --- | --- | --- | --- | --- | --- | --- | --- | --- |
|  |  |  |  | **No.** | **%** | **No.** | **%** |  | **p-value** |  |
|  | Age (median [range], years) | |  | 59 (86-26) | | 60 (90-20) | |  | 0.270 |  |
|  | Sex | Male |  | 251 | 70.5 | 533 | 62.3 |  | 0.007 |  |
|  |  | Female |  | 105 | 29.5 | 322 | 37.7 |  |  |  |
|  | Stage* | 0 |  | 36 | 10.1 | 141 | 16.5 |  | 0.227 |  |
|  |  | I |  | 110 | 30.9 | 246 | 28.8 |  |  |  |
|  |  | II |  | 116 | 32.6 | 231 | 27.0 |  |  |  |
|  |  | III |  | 94 | 26.4 | 237 | 27.7 |  |  |  |
|  | Tumor location | Upper-Middle |  | 192 | 53.9 | 527 | 61.6 |  | 0.013 |  |
|  |  | Lower |  | 164 | 46.1 | 328 | 38.4 |  |  |  |
|  | CCRT regimen | Capecitabine |  | 15 | 4.2 | 667 | 78.0 |  | <0.001 |  |
|  |  | FL or others |  | 341 | 95.8 | 188 | 22.0 |  |  |  |
|  | Adjuvant chemotherapy | Yes |  | 197 | 55.3 | 473 | 55.3 |  | 0.996 |  |
|  |  | No |  | 159 | 44.7 | 382 | 44.7 |  |  |  |

* Pathologic staging
*Abbreviations:* MDT, Multidisciplinary team; CCRT, concurrent chemoradiotherapy; FL, 5-Fluorouracil and Leucovorin

**Supplementary Table 2.** Comparison of characteristics between the pre-MDT clinic cohort and the MDT clinic cohort in patients with recurrences

|  |  | **Pre-MDT clinic cohort** | | **MDT clinic cohort** | |  |
| --- | --- | --- | --- | --- | --- | --- |
|  |  | **No.** | **%** | **No.** | **%** | **p-value** |
| **Before recurrences** |  |  |  |  |  |  |
| Age at diagnosis (years, mean±SD) | | 58.1±1.2 | | 58.0±0.9 | | 0.948 |
| Sex | Male | 67 | 77 | 122 | 62.9 | 0.02 |
|  | Female | 20 | 23 | 72 | 37.1 |  |
| icT stage | T1-2 | 5 | 5.7 | 12 | 6.3 | 0.863 |
|  | T3-4 | 82 | 94.3 | 179 | 93.7 |  |
| icN stage | N0 | 16 | 18.4 | 36 | 18.6 | 0.974 |
|  | N+ | 71 | 81.6 | 158 | 81.4 |  |
| ypT stage | T0-2 | 29 | 33.3 | 48 | 24.7 | 0.135 |
|  | T3-4 | 58 | 66.7 | 146 | 75.3 |  |
| ypN stage | N0 | 45 | 51.7 | 97 | 50 | 0.789 |
|  | N1-2 | 42 | 48.3 | 97 | 50 |  |
| pCR | No | 82 | 94.3 | 188 | 96.9 | 0.289 |
|  | Yes | 5 | 5.7 | 6 | 3.1 |  |
| Tumor location | Upper | 4 | 4.6 | 29 | 14.9 | 0.013 |
|  | Middle-lower | 83 | 95.4 | 165 | 85.1 |  |
| Tumor differentiation | MD~PD | 65 | 75.6 | 160 | 82.9 | 0.153 |
|  | WD | 21 | 24.4 | 33 | 17.1 |  |
| RT modality | 3DCRT | 87 | 100 | 110 | 56.7 | <0.001 |
|  | IMRT | 0 | 0 | 84 | 43.3 |  |
| CCRT regimen | FL | 54 | 62.1 | 44 | 22.7 | <0.001 |
|  | Xeloda | 4 | 4.6 | 149 | 76.8 |  |
|  | others | 29 | 33.3 | 1 | 0.5 |  |
| Adjuvant chemotherapy regimen | Not performed | 25 | 28.7 | 68 | 35.1 | <0.001 |
|  | FL | 48 | 55.2 | 27 | 13.9 |  |
|  | FOLFOX | 6 | 6.9 | 65 | 33.5 |  |
|  | Xeloda | 8 | 9.2 | 32 | 16.5 |  |
|  | XELOX | 0 | 0 | 2 | 1 |  |
| **After recurrences** |  |  |  |  |  |  |
| Age at recurrence (years, mean±SD) |  | 59.9±1.2 | | 59.7±0.9 | | 0.897 |
| Cumulative number of RT session per patient | | 0.9±0.1 | | 0.8±0.1 | | 0.76 |
| Cumulative number of surgery session per patient | | 1.1±0.1 | | 1.0±0.1 | | 0.64 |
| Cumulative number of surgery ± RT session per patient | | 1.9±0.2 | | 1.8±0.1 | | 0.607 |
| Cumulative number of changes in systemic therapy | | 1.9±0.1 | | 1.9±0.1 | | 0.684 |
| FOLFOX | Not performed | 35 | 40.2 | 73 | 37.6 | 0.679 |
|  | Performed | 52 | 59.8 | 121 | 62.4 |  |
| FOLFIRI | Not performed | 40 | 46 | 69 | 35.6 | 0.098 |
|  | Performed | 47 | 54 | 125 | 64.4 |  |
| FOLFOX or FOLFIRI | Not performed | 18 | 20.7 | 37 | 19.1 | 0.752 |
|  | Performed | 69 | 79.3 | 157 | 80.9 |  |
| Xeloda | Not performed | 44 | 50.6 | 120 | 61.9 | 0.076 |
|  | Performed | 43 | 49.4 | 74 | 38.1 |  |
| Lonsurf | Not performed | 87 | 100 | 185 | 95.4 | 0.041 |
|  | Performed | 0 | 0 | 9 | 4.6 |  |
| Targeted agent | Not performed | 63 | 72.4 | 56 | 28.9 | <0.001 |
|  | Performed | 24 | 27.6 | 138 | 71.1 |  |

Abbreviations: MDT, Multidisciplinary team; CR, complete response; MD, moderately differentiated; PD, poorly differentiated; WD, well differentiated; 3DCRT, 3-dimensional conformal radiotherapy; IMRT, intensity-modulated radiotherapy; RT, radiotherapy; CCRT, concurrent chemoradiotherapy; FL, 5-Fluorouracil and Leucovorin; FOLFOX, oxaliplatin, infusional fluorouracil, and leucovorin; FOLFIRI, irinotecan, infusional fluorouracil, and leucovorin

**Supplementary Table 3**. Practice patterns of local therapy (radiotherapy and/or surgery) usage according to the year of recurrence in MDT-pt (-) group

|  |  | **Cumulative number of surgery ± RT session per patient** | **Cumulative number of RT session per patient** | **Cumulative number of surgery session per patient** |
| --- | --- | --- | --- | --- |
| The year of recurrence | 2007 | 2.0 | 1.5 | 0.5 |
|  | 2008 | 1.6 | 0.9 | 0.7 |
|  | 2009 | 1.8 | 0.6 | 1.1 |
|  | 2010 | 2.1 | 1.3 | 0.8 |
|  | 2011 | 1.6 | 0.7 | 0.9 |
|  | 2012 | 2.1 | 0.9 | 1.3 |
|  | 2013 | 0.9 | 0.4 | 0.5 |
|  | 2014 | 1.9 | 0.4 | 1.5 |
|  | 2015 | 1.6 | 0.7 | 0.9 |
|  | 2016 | 1.9 | 0.8 | 1.1 |
|  | 2017 | 1.6 | 0.5 | 1.1 |
|  | 2018 | 1.3 | 0.8 | 0.6 |
|  | 2019 | 1.1 | 0.6 | 0.5 |
|  | 2020 | 1.3 | 0.5 | 0.8 |

Abbreviations: RT, radiotherapy
